# Crystal structure of ethyl 2-(3-amino-5-oxo-2-tosyl-2,5-di­hydro-1*H*-pyrazol-1-yl)acetate

**DOI:** 10.1107/S2056989021004795

**Published:** 2021-05-14

**Authors:** Nadia H. Metwally, Galal H. Elgemeie, Peter G. Jones

**Affiliations:** a Chemistry Department, Faculty of Science, Cairo University, Giza, Egypt; b Chemistry Department, Faculty of Science, Helwan University, Cairo, Egypt; c Institut für Anorganische und Analytische Chemie, Technische Universität Braunschweig, Hagenring 30, D-38106 Braunschweig, Germany

**Keywords:** pyrazole, tos­yl, hydrogen bond, crystal structure

## Abstract

In the title compound, C_14_H_17_N_3_O_5_S, the five-membered ring is essentially planar. The substituents at the nitro­gen atoms subtend a C—N—N—S torsion angle of −95.52 (6)°. The amino group forms an intra­molecular hydrogen bond to a sulfonyl oxygen atom; two inter­molecular hydrogen bonds from the amino group, to the other S=O group and to the oxo substituent, form a layer structure parallel to the *ab* plane. The structure determination confirms that the title compound is *N*- rather than *O*-alkyl­ated.

## Abstract 2

The *N*-substituents lie on opposite sides of the pyrazole ring. Inter­molecular hydrogen bonds from the amino group to an S=O group and to the oxo substituent lead to a layer structure.

## Chemical context   

Recently we have been attempting to develop synthetic strategies for heterocyclic ring systems containing *N*-sulfonyl­amino- and *N*-sulfonyl moieties. The products may be biologically active, displaying for instance anti-viral activity (Azzam *et al.*, 2017 ▸, 2019 ▸, 2020 ▸; Zhu *et al.*, 2013 ▸; Elgemeie *et al.*, 2017 ▸, 2019 ▸). Also, some of our reported *N*-aryl­sulfonyl­pyrazole derivatives (Elgemeie *et al.*, 1998 ▸, 2013 ▸; Elgemeie & Hanfy, 1999 ▸) proved to be inhibitors of the NS2B-NS3 virus and the enzyme cathepsin B16 (Myers *et al.*, 2007 ▸; Sidique *et al.*, 2009 ▸). In a continuation of our research investigating new approaches to other new derivatives of *N*-sulfonyl­pyrazoles, seeking various scaffolds for use as encouraging chemotherapeutics (Zhang *et al.*, 2020 ▸; Elgemeie & Jones, 2002 ▸), we have now synthesized the N1-substituted derivative of *N*-sulfonyl­pyrazole **1** (the structure of which we have reported; Elgemeie *et al.*, 2013 ▸).
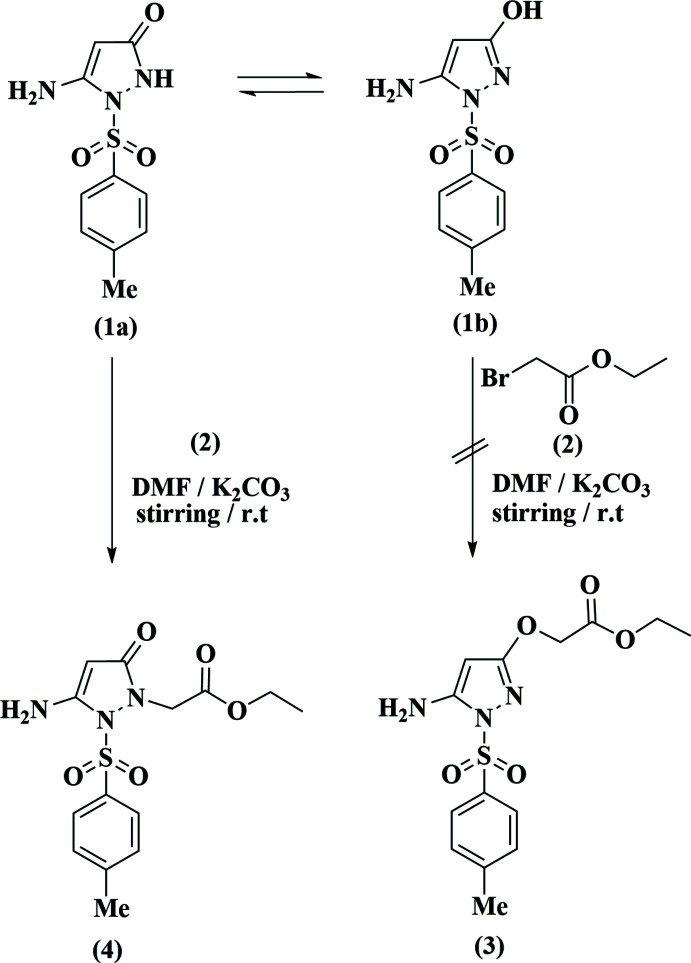


The reaction of **1** with ethyl bromo­acetate **2** in dry *N*,*N*-di­methyl­formamide containing anhydrous potassium carbonate at room temperature afforded a product for which two possible isomeric structures, the *N*-alkyl­ated or *O*-alkyl­ated *N*-sulfonyl­pyrazoles **3** or **4**, were feasible. The ^1^H NMR spectrum of the product showed four singlet signals at δ = 2.41, 4.31, 4.40 and 7.15 ppm assigned to CH_3_, pyrazole-CH, CH_2_ and NH_2_ protons, along with triplet and quartet signals at δ = 1.17 and 4.09 ppm, assigned to ethyl groups. The spectroscopic data cannot differentiate between structures **3** and **4**. We therefore determined the X-ray structure of this product, which proved to be the *N*-alkyl­ated-*N*-sulfonyl­pyrazole **4**.

## Structural commentary   

**Figure 1:**
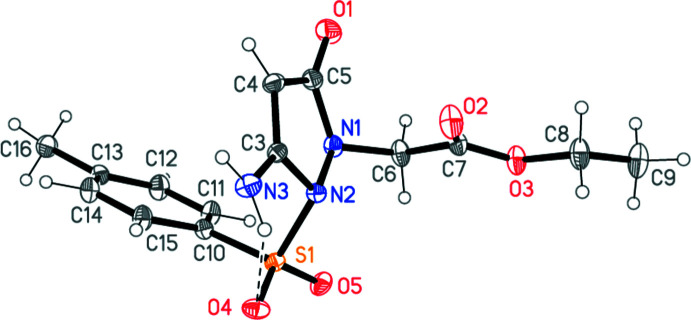
The structure of compound **4** in the crystal. Ellipsoids represent 50% probability levels. The dashed line indicates an intra­molecular hydrogen bond.

The mol­ecule of compound **4** is shown in Fig. 1 ▸. Mol­ecular dimensions, a selection of which are presented in Table 1 ▸, may be considered normal (*e.g*. the N1—N2 bond length corres­ponds to a single bond and these atoms display a pyramidal geometry). The substituents S1 and C6 of the five-membered ring, which is effectively planar (r.m.s. deviation 0.026 Å) lie significantly outside the ring plane [by 1.2344 (8) and 0.8468 (19) Å, respectively] in opposite directions; the corresponding torsion angle C6—N1—N2—S1 is −95.52 (6)°. The side chain at N1 exhibits an extended conformation. An intra­molecular hydrogen bond is formed from the amino group to the sulfonyl oxygen atom O4 (Table 2 ▸). The ring planes subtend an inter­planar angle of 57.01 (3)°.

## Supra­molecular features   

**Figure 2:**
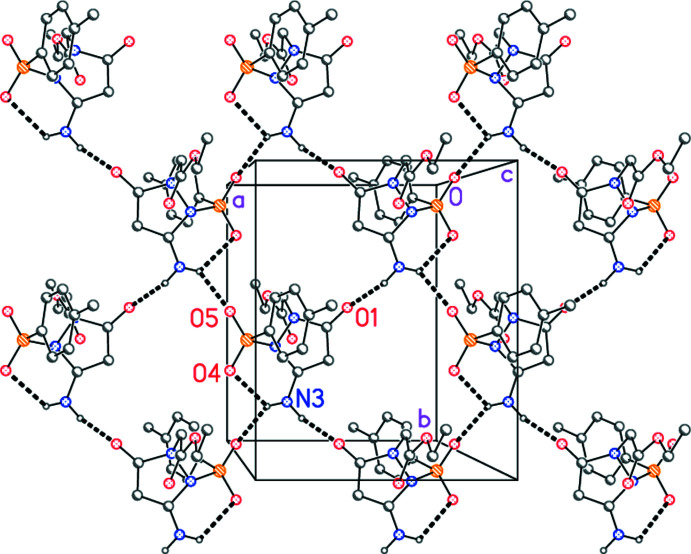
Packing diagram of compound **4** viewed perpendicular to the *ab* plane in the region *z* ≃ 0.25. Dashed lines indicate hydrogen bonds. Hydrogen atoms not involved in hydrogen bonding are omitted for clarity.

The mol­ecules of **4** are linked by two classical hydrogen bonds, from the NH_2_ hydrogen atoms H01 and H02 to the acceptors O5=S1 and O1=C5, to form layers parallel to the *ab* plane (Fig. 2 ▸, Table 2 ▸). The hydrogen atom H02 is thus involved in a three-centre hydrogen bond, taking the above-mentioned intra­molecular inter­action into account. The additional ‘weak’ inter­actions H6*B*⋯O4 (within the layers; operator −*x* + 2, *y* − 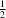, −*z* + 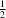) and H8*B*⋯O1 (between layers; operator −*x* + 1, −*y* + 1, −*z*) are not shown in the Figure. The shortest distance between ring centroids is 3.97 Å for the ring C10–C15 (operator 2 − *x*, 1 − *y*, 1 − *z*).

## Database survey   

A database search (CSD Version 5.41) for the same ring system as in **4**, and bearing the same substituents at C5 (oxo) and C3 (amino), gave eight hits involving uncharged species. However, none of these was substituted at both ring nitro­gen atoms. Two (our previous structures: Elgemeie *et al.*, 1998 ▸, 2013 ▸) have a hydrogen atom at N1, while the other six have a hydrogen at N2; the other *N*-substituents are 9-thioxanthenyl (DOJKIW; Kimura, 1986 ▸), C(=S)NHEt (LUPDUW; Pitucha *et al.*, 2010 ▸), C(=O)NHCH_2_COOEt (MAVJUK) and C(=O)NH-^*n*^Bu (MAVKAX; Pitucha *et al.*, 2011 ▸), C(=O)NHCH(Ph)CH_3_ (TIRVAT; Kaczor *et al.*, 2013 ▸) and C(O)NH-1-naphthyl (VOQGOZ; Kaczor *et al.*, 2014 ▸). It is notable that the *X*—N—N—X (*X* = H or substituent atom) torsion angles are very variable; in four cases the absolute value lies between 0 and 11°, whereas for the bulky subs­tituents in DOJKIW and VOQGOZ the values are 63.7 and 32.1°, respectively.

## Synthesis and crystallization   

A mixture of 5-amino-1-tosyl-1,2-di­hydro-3*H*-pyrazol-3-one **1** (0.01 mol), ethyl bromo­acetate **2** (0.01 mol) and anhydrous potassium carbonate (0.01 mol) in *N*,*N*-di­methyl­formamide (5 mL) was stirred at room temperature for 3 h. The mixture was poured onto ice–water; the solid thus formed was filtered off and recrystallized from ethanol to give pale yellow crystals in 64% yield, m.p. = 415–416 K. IR (KBr, cm^−1^): ν 3460, 3297 (NH_2_), 1752 (ester C=O), 1700 (ring C=O); ^1^H NMR (DMSO-*d*_6_): δ = 1.17 (*t*, 3H, *J* = 7.2 Hz, CH_3_), 2.41 (*s*, 3H, CH_3_), 4.09 (*q*, 2H, *J* = 7.2 Hz, CH_2_), 4.31 (*s*, 1H, CH pyrazole), 4.40 (*s*, 2H, CH_2_), 7.15 (*s*, 2H, NH_2_), 7.45 (*d*, 2H, *J* = 8.4 Hz, Ar), 7.73 (*d*, 2H, *J* = 8.4 Hz, Ar). Analysis: calculated C_14_H_17_N_3_O_5_S (339.36); C, 49.55; H, 5.05; N, 12.38; S, 9.45. Found: C, 49.38; H, 5.23; N, 12.59; S, 9.27%.

## Refinement   

Crystal data, data collection and structure refinement details are summarized in Table 3 ▸. The hydrogen atoms of the NH_2_ group were refined freely. The methyl groups were refined as idealized rigid groups allowed to rotate but not tip (AFIX 137; C—H = 0.98 Å, H—C—H = 109.5°). Other hydrogens were included using a riding model starting from calculated positions (C—H_aromatic_ = 0.95, C—H_methyl­ene_ = 0.99 Å). The *U*_iso_(H) values were fixed at 1.5 (for the methyl H) or 1.2 times the equivalent *U*_eq_ value of the parent carbon atoms.

## Supplementary Material

Crystal structure: contains datablock(s) I, global. DOI: 10.1107/S2056989021004795/zl5011sup1.cif

Structure factors: contains datablock(s) I. DOI: 10.1107/S2056989021004795/zl5011Isup2.hkl

Supporting information file. DOI: 10.1107/S2056989021004795/zl5011Isup3.cml

CCDC reference: 2082046

Additional supporting information:  crystallographic information; 3D view; checkCIF report

## Figures and Tables

**Table 1:** Selected geometric parameters (Å, °)

N1—C5	1.4157 (9)	N2—S1	1.7154 (6)
N1—N2	1.4296 (8)	C3—C4	1.3661 (9)
N1—C6	1.4549 (9)	C4—C5	1.4203 (10)
N2—C3	1.4273 (8)		
			
C5—N1—N2	107.67 (5)	C4—C3—N2	110.21 (6)
C5—N1—C6	117.34 (6)	C3—C4—C5	107.94 (6)
N2—N1—C6	115.11 (5)	O1—C5—N1	120.24 (7)
C3—N2—N1	105.89 (5)	O1—C5—C4	131.82 (7)
C3—N2—S1	116.65 (4)	N1—C5—C4	107.90 (6)
N1—N2—S1	109.25 (4)		
			
C6—N1—N2—S1	−95.52 (6)	C6—C7—O3—C8	−179.18 (6)
N1—C6—C7—O3	170.80 (6)	C9—C8—O3—C7	−168.27 (7)

**Table 2:** Hydrogen-bond geometry (Å, °)

*D*—H⋯*A*	*D*—H	H⋯*A*	*D*⋯*A*	*D*—H⋯*A*
N3—H01⋯O1^i^	0.913 (13)	1.897 (13)	2.7884 (8)	164.7 (12)
N3—H02⋯O4	0.861 (13)	2.394 (13)	2.8291 (8)	111.8 (10)
N3—H02⋯O5^ii^	0.861 (13)	2.294 (13)	3.0139 (8)	141.3 (12)
C6—H6*B*⋯O4^iii^	0.99	2.50	3.2642 (9)	133
C8—H8*B*⋯O1^iv^	0.99	2.43	3.2663 (11)	142

Symmetry codes: (i) -x+1, y+{\script{1\over 2}}, -z+{\script{1\over 2}}; (ii) -x+2, y+{\script{1\over 2}}, -z+{\script{1\over 2}}; (iii) -x+2, y-{\script{1\over 2}}, -z+{\script{1\over 2}}; (iv) -x+1, -y+1, -z.

**Table 3:** Experimental details

Crystal data	
Chemical formula	C_14_H_17_N_3_O_5_S
*M*_r_	339.36
Crystal system, space group	Monoclinic, *P*2_1_/*c*
Temperature (K)	100
*a*, *b*, *c* (Å)	9.1398 (2), 11.1525 (2), 16.3795 (3)
β (°)	97.081 (2)
*V* (Å^3^)	1656.85 (6)
*Z*	4
Radiation type	Mo *K*α
μ (mm^−1^)	0.22
Crystal size (mm)	0.24 × 0.20 × 0.08

Data collection	
Diffractometer	XtaLAB Synergy, Single source at offset/far, HyPix
Absorption correction	Multi-scan (*CrysAlis PRO*; Rigaku OD, 2021 ▸)
*T*_min_, *T*_max_	0.805, 1.000
No. of measured, independent and observed [*I* > 2σ(*I*)] reflections	128543, 7466, 6502
*R*_int_	0.040
(sin θ/λ)_max_ (Å^−1^)	0.825

Refinement	
*R*[*F* ^2^ > 2σ(*F* ^2^)], *wR*(*F* ^2^), *S*	0.031, 0.094, 1.05
No. of reflections	7466
No. of parameters	218
H-atom treatment	H atoms treated by a mixture of independent and constrained refinement
Δρ_max_, Δρ_min_ (e Å^−3^)	0.58, −0.30

Computer programs: *CrysAlis PRO* (Rigaku OD, 2021 ▸), *SHELXT* (Sheldrick, 2015*a* ▸), *SHELXL2018/3* (Sheldrick, 2015*b* ▸) and *XP* (Siemens, 1994 ▸).

## supplementary crystallographic information

### Crystal data

**Table:**

C_14_H_17_N_3_O_5_S	*F*(000) = 712
*M**_r_* = 339.36	*D*_x_ = 1.360 Mg m^−^^3^
Monoclinic, *P*2_1_/*c*	Mo *K*α radiation, λ = 0.71073 Å
*a* = 9.1398 (2) Å	Cell parameters from 78897 reflections
*b* = 11.1525 (2) Å	θ = 2.2–36.1°
*c* = 16.3795 (3) Å	µ = 0.22 mm^−^^1^
β = 97.081 (2)°	*T* = 100 K
*V* = 1656.85 (6) Å^3^	Tablet, colourless
*Z* = 4	0.24 × 0.20 × 0.08 mm

### Data collection

**Table:**

XtaLAB Synergy, Single source at offset/far, HyPix diffractometer	7466 independent reflections
Radiation source: micro-focus sealed X-ray tube	6502 reflections with *I* > 2σ(*I*)
Detector resolution: 10.0000 pixels mm^-1^	*R*_int_ = 0.040
ω scans	θ_max_ = 35.9°, θ_min_ = 2.2°
Absorption correction: multi-scan (CrysAlisPro; Rigaku OD, 2021)	*h* = −14→15
*T*_min_ = 0.805, *T*_max_ = 1.000	*k* = −17→18
128543 measured reflections	*l* = −26→26

### Refinement

**Table:**

Refinement on *F*^2^	Primary atom site location: dual
Least-squares matrix: full	Secondary atom site location: difference Fourier map
*R*[*F*^2^ > 2σ(*F*^2^)] = 0.031	Hydrogen site location: mixed
*wR*(*F*^2^) = 0.094	H atoms treated by a mixture of independent and constrained refinement
*S* = 1.05	*w* = 1/[σ^2^(*F*_o_^2^) + (0.0543*P*)^2^ + 0.3217*P*] where *P* = (*F*_o_^2^ + 2*F*_c_^2^)/3
7466 reflections	(Δ/σ)_max_ = 0.001
218 parameters	Δρ_max_ = 0.58 e Å^−^^3^
0 restraints	Δρ_min_ = −0.30 e Å^−^^3^

### Special details

**Table:**

Geometry. All esds (except the esd in the dihedral angle between two l.s. planes) are estimated using the full covariance matrix. The cell esds are taken into account individually in the estimation of esds in distances, angles and torsion angles; correlations between esds in cell parameters are only used when they are defined by crystal symmetry. An approximate (isotropic) treatment of cell esds is used for estimating esds involving l.s. planes. Least-squares planes (x,y,z in crystal coordinates) and deviations from them (* indicates atom used to define plane) - 0.4791 (0.0031) x - 2.3604 (0.0036) y + 15.9693 (0.0013) z = 2.0024 (0.0032) * 0.0342 (0.0004) N1 * -0.0209 (0.0004) N2 * -0.0003 (0.0004) C3 * 0.0215 (0.0004) C4 * -0.0345 (0.0004) C5 -0.8468 (0.0010) C6 0.0094 (0.0011) N3 -0.1626 (0.0011) O1 1.2344 (0.0008) S1 Rms deviation of fitted atoms = 0.0255 7.3350 (0.0016) x - 0.2526 (0.0037) y + 8.0701 (0.0042) z = 9.4209 (0.0022) Angle to previous plane (with approximate esd) = 57.006 ( 0.025 ) * -0.0031 (0.0005) C10 * -0.0009 (0.0005) C11 * 0.0057 (0.0006) C12 * -0.0065 (0.0005) C13 * 0.0025 (0.0005) C14 * 0.0022 (0.0005) C15 -0.0668 (0.0010) S1 -0.0236 (0.0012) C16 Rms deviation of fitted atoms = 0.0040

### Fractional atomic coordinates and isotropic or equivalent isotropic displacement parameters (Å^2^)

**Table:**

	*x*	*y*	*z*	*U*_iso_*/*U*_eq_	
N1	0.71924 (7)	0.51318 (5)	0.22496 (4)	0.01525 (10)	
N2	0.80727 (6)	0.61874 (5)	0.23976 (3)	0.01313 (9)	
C3	0.70693 (7)	0.71323 (6)	0.25200 (4)	0.01349 (10)	
C4	0.56556 (7)	0.67098 (6)	0.24288 (4)	0.01709 (11)	
H4	0.479736	0.716052	0.249768	0.021*	
C5	0.56954 (8)	0.54779 (6)	0.22128 (4)	0.01753 (12)	
O1	0.47057 (7)	0.47513 (6)	0.19955 (4)	0.02624 (12)	
N3	0.75894 (7)	0.82301 (5)	0.27039 (4)	0.01797 (11)	
H01	0.6933 (15)	0.8832 (12)	0.2762 (8)	0.029 (3)*	
H02	0.8476 (15)	0.8402 (12)	0.2619 (8)	0.029 (3)*	
C6	0.75926 (9)	0.43604 (6)	0.15959 (4)	0.01874 (12)	
H6A	0.692061	0.366114	0.153801	0.022*	
H6B	0.860733	0.405756	0.174982	0.022*	
C7	0.75148 (8)	0.50043 (6)	0.07754 (4)	0.01746 (12)	
C8	0.81058 (10)	0.48513 (7)	−0.05858 (5)	0.02349 (14)	
H8A	0.884230	0.550504	−0.056465	0.028*	
H8B	0.712342	0.518726	−0.078401	0.028*	
O2	0.70022 (8)	0.59873 (5)	0.06371 (4)	0.02588 (12)	
O3	0.80980 (7)	0.43200 (5)	0.02284 (3)	0.02152 (11)	
C9	0.84936 (12)	0.38700 (8)	−0.11506 (5)	0.02858 (17)	
H9A	0.856512	0.420399	−0.169766	0.043*	
H9B	0.772657	0.325136	−0.119119	0.043*	
H9C	0.944187	0.351494	−0.093111	0.043*	
S1	0.94519 (2)	0.59188 (2)	0.31853 (2)	0.01400 (4)	
O4	1.02319 (6)	0.70299 (5)	0.33031 (4)	0.02030 (10)	
O5	1.01852 (6)	0.48758 (5)	0.29250 (3)	0.01987 (10)	
C10	0.85810 (7)	0.55582 (6)	0.40445 (4)	0.01440 (10)	
C11	0.83563 (9)	0.43557 (6)	0.42138 (4)	0.01902 (12)	
H11	0.869968	0.374683	0.387917	0.023*	
C12	0.76214 (9)	0.40563 (7)	0.48806 (5)	0.02152 (13)	
H12	0.747436	0.323625	0.500452	0.026*	
C13	0.70971 (8)	0.49456 (7)	0.53699 (4)	0.01845 (12)	
C14	0.73476 (9)	0.61451 (7)	0.51909 (5)	0.02110 (13)	
H14	0.700833	0.675486	0.552637	0.025*	
C15	0.80858 (9)	0.64644 (6)	0.45295 (4)	0.01932 (12)	
H15	0.824955	0.728372	0.441058	0.023*	
C16	0.62816 (9)	0.46037 (9)	0.60792 (5)	0.02581 (15)	
H16A	0.699167	0.440717	0.655867	0.039*	
H16B	0.565744	0.390460	0.592686	0.039*	
H16C	0.566462	0.527646	0.621388	0.039*	

### Atomic displacement parameters (Å^2^)

**Table:**

	*U*^11^	*U*^22^	*U*^33^	*U*^12^	*U*^13^	*U*^23^
N1	0.0188 (2)	0.0120 (2)	0.0151 (2)	−0.00278 (17)	0.00269 (18)	−0.00152 (17)
N2	0.0136 (2)	0.0110 (2)	0.0147 (2)	−0.00006 (16)	0.00156 (16)	−0.00001 (16)
C3	0.0135 (2)	0.0126 (2)	0.0146 (2)	0.00057 (18)	0.00261 (18)	0.00023 (18)
C4	0.0130 (2)	0.0188 (3)	0.0197 (3)	−0.0009 (2)	0.0032 (2)	−0.0015 (2)
C5	0.0173 (3)	0.0193 (3)	0.0161 (3)	−0.0050 (2)	0.0029 (2)	−0.0004 (2)
O1	0.0242 (3)	0.0283 (3)	0.0262 (3)	−0.0140 (2)	0.0030 (2)	−0.0038 (2)
N3	0.0167 (2)	0.0115 (2)	0.0261 (3)	−0.00030 (18)	0.0040 (2)	−0.00131 (19)
C6	0.0296 (3)	0.0122 (2)	0.0145 (3)	0.0014 (2)	0.0028 (2)	−0.0007 (2)
C7	0.0228 (3)	0.0151 (3)	0.0142 (3)	0.0014 (2)	0.0011 (2)	−0.0009 (2)
C8	0.0373 (4)	0.0182 (3)	0.0157 (3)	0.0052 (3)	0.0060 (3)	0.0023 (2)
O2	0.0418 (3)	0.0172 (2)	0.0188 (2)	0.0097 (2)	0.0042 (2)	0.00153 (18)
O3	0.0327 (3)	0.0179 (2)	0.0144 (2)	0.0073 (2)	0.00453 (19)	0.00122 (17)
C9	0.0462 (5)	0.0220 (3)	0.0196 (3)	0.0069 (3)	0.0121 (3)	0.0011 (3)
S1	0.01253 (7)	0.01380 (7)	0.01577 (7)	0.00071 (4)	0.00218 (5)	0.00157 (5)
O4	0.0162 (2)	0.0189 (2)	0.0251 (2)	−0.00522 (17)	−0.00029 (18)	0.00314 (18)
O5	0.0189 (2)	0.0205 (2)	0.0210 (2)	0.00769 (18)	0.00564 (18)	0.00221 (18)
C10	0.0165 (3)	0.0128 (2)	0.0139 (2)	0.00007 (19)	0.00173 (19)	−0.00002 (19)
C11	0.0266 (3)	0.0136 (3)	0.0178 (3)	0.0004 (2)	0.0068 (2)	0.0007 (2)
C12	0.0293 (4)	0.0174 (3)	0.0188 (3)	−0.0025 (2)	0.0070 (3)	0.0021 (2)
C13	0.0172 (3)	0.0239 (3)	0.0141 (3)	−0.0017 (2)	0.0014 (2)	−0.0004 (2)
C14	0.0251 (3)	0.0207 (3)	0.0182 (3)	0.0013 (2)	0.0052 (2)	−0.0039 (2)
C15	0.0255 (3)	0.0142 (3)	0.0187 (3)	0.0004 (2)	0.0045 (2)	−0.0026 (2)
C16	0.0223 (3)	0.0393 (4)	0.0165 (3)	−0.0045 (3)	0.0053 (2)	0.0009 (3)

### Geometric parameters (Å, º)

**Table:**

N1—C5	1.4157 (9)	C13—C14	1.3944 (11)
N1—N2	1.4296 (8)	C13—C16	1.5046 (10)
N1—C6	1.4549 (9)	C14—C15	1.3913 (11)
N2—C3	1.4273 (8)	C4—H4	0.9500
N2—S1	1.7154 (6)	N3—H01	0.913 (13)
C3—N3	1.3343 (8)	N3—H02	0.861 (13)
C3—C4	1.3661 (9)	C6—H6A	0.9900
C4—C5	1.4203 (10)	C6—H6B	0.9900
C5—O1	1.2337 (8)	C8—H8A	0.9900
C6—C7	1.5177 (10)	C8—H8B	0.9900
C7—O2	1.2027 (8)	C9—H9A	0.9800
C7—O3	1.3367 (9)	C9—H9B	0.9800
C8—O3	1.4601 (9)	C9—H9C	0.9800
C8—C9	1.5033 (11)	C11—H11	0.9500
S1—O4	1.4308 (6)	C12—H12	0.9500
S1—O5	1.4334 (5)	C14—H14	0.9500
S1—C10	1.7470 (7)	C15—H15	0.9500
C10—C11	1.3899 (9)	C16—H16A	0.9800
C10—C15	1.3950 (9)	C16—H16B	0.9800
C11—C12	1.3914 (10)	C16—H16C	0.9800
C12—C13	1.3966 (11)		
			
C5—N1—N2	107.67 (5)	C3—C4—H4	126.0
C5—N1—C6	117.34 (6)	C5—C4—H4	126.0
N2—N1—C6	115.11 (5)	C3—N3—H01	118.5 (8)
C3—N2—N1	105.89 (5)	C3—N3—H02	119.0 (9)
C3—N2—S1	116.65 (4)	H01—N3—H02	119.8 (12)
N1—N2—S1	109.25 (4)	N1—C6—H6A	109.1
N3—C3—C4	130.31 (6)	C7—C6—H6A	109.1
N3—C3—N2	119.47 (6)	N1—C6—H6B	109.1
C4—C3—N2	110.21 (6)	C7—C6—H6B	109.1
C3—C4—C5	107.94 (6)	H6A—C6—H6B	107.8
O1—C5—N1	120.24 (7)	O3—C8—H8A	110.3
O1—C5—C4	131.82 (7)	C9—C8—H8A	110.3
N1—C5—C4	107.90 (6)	O3—C8—H8B	110.3
N1—C6—C7	112.54 (6)	C9—C8—H8B	110.3
O2—C7—O3	124.92 (7)	H8A—C8—H8B	108.5
O2—C7—C6	124.94 (7)	C8—C9—H9A	109.5
O3—C7—C6	110.15 (6)	C8—C9—H9B	109.5
O3—C8—C9	107.14 (6)	H9A—C9—H9B	109.5
C7—O3—C8	115.33 (6)	C8—C9—H9C	109.5
O4—S1—O5	119.96 (4)	H9A—C9—H9C	109.5
O4—S1—N2	104.95 (3)	H9B—C9—H9C	109.5
O5—S1—N2	104.19 (3)	C10—C11—H11	120.5
O4—S1—C10	111.08 (3)	C12—C11—H11	120.5
O5—S1—C10	109.21 (3)	C11—C12—H12	119.6
N2—S1—C10	106.30 (3)	C13—C12—H12	119.6
C11—C10—C15	121.27 (6)	C15—C14—H14	119.4
C11—C10—S1	118.43 (5)	C13—C14—H14	119.4
C15—C10—S1	120.27 (5)	C14—C15—H15	120.6
C10—C11—C12	119.05 (6)	C10—C15—H15	120.6
C11—C12—C13	120.87 (7)	C13—C16—H16A	109.5
C14—C13—C12	118.93 (6)	C13—C16—H16B	109.5
C14—C13—C16	121.02 (7)	H16A—C16—H16B	109.5
C12—C13—C16	120.05 (7)	C13—C16—H16C	109.5
C15—C14—C13	121.14 (7)	H16A—C16—H16C	109.5
C14—C15—C10	118.72 (7)	H16B—C16—H16C	109.5
			
C5—N1—N2—C3	5.12 (7)	C3—N2—S1—O4	−58.13 (5)
C6—N1—N2—C3	138.08 (6)	N1—N2—S1—O4	−178.12 (4)
C5—N1—N2—S1	131.52 (5)	C3—N2—S1—O5	174.95 (5)
C6—N1—N2—S1	−95.52 (6)	N1—N2—S1—O5	54.96 (5)
N1—N2—C3—N3	177.46 (6)	C3—N2—S1—C10	59.64 (5)
S1—N2—C3—N3	55.69 (7)	N1—N2—S1—C10	−60.35 (5)
N1—N2—C3—C4	−1.86 (7)	O4—S1—C10—C11	−151.48 (6)
S1—N2—C3—C4	−123.63 (5)	O5—S1—C10—C11	−16.96 (7)
N3—C3—C4—C5	178.65 (7)	N2—S1—C10—C11	94.90 (6)
N2—C3—C4—C5	−2.13 (8)	O4—S1—C10—C15	30.51 (7)
N2—N1—C5—O1	171.51 (6)	O5—S1—C10—C15	165.03 (6)
C6—N1—C5—O1	39.75 (9)	N2—S1—C10—C15	−83.11 (6)
N2—N1—C5—C4	−6.48 (7)	C15—C10—C11—C12	0.06 (11)
C6—N1—C5—C4	−138.24 (6)	S1—C10—C11—C12	−177.93 (6)
C3—C4—C5—O1	−172.33 (8)	C10—C11—C12—C13	0.78 (12)
C3—C4—C5—N1	5.35 (8)	C11—C12—C13—C14	−1.31 (12)
C5—N1—C6—C7	69.88 (8)	C11—C12—C13—C16	178.92 (7)
N2—N1—C6—C7	−58.40 (8)	C12—C13—C14—C15	1.01 (12)
N1—C6—C7—O2	−9.23 (11)	C16—C13—C14—C15	−179.22 (7)
N1—C6—C7—O3	170.80 (6)	C13—C14—C15—C10	−0.20 (11)
O2—C7—O3—C8	0.85 (12)	C11—C10—C15—C14	−0.35 (11)
C6—C7—O3—C8	−179.18 (6)	S1—C10—C15—C14	177.60 (6)
C9—C8—O3—C7	−168.27 (7)		

### Hydrogen-bond geometry (Å, º)

**Table:**

*D*—H···*A*	*D*—H	H···*A*	*D*···*A*	*D*—H···*A*
N3—H01···O1^i^	0.913 (13)	1.897 (13)	2.7884 (8)	164.7 (12)
N3—H02···O4	0.861 (13)	2.394 (13)	2.8291 (8)	111.8 (10)
N3—H02···O5^ii^	0.861 (13)	2.294 (13)	3.0139 (8)	141.3 (12)
C6—H6*B*···O4^iii^	0.99	2.50	3.2642 (9)	133
C8—H8*B*···O1^iv^	0.99	2.43	3.2663 (11)	142

Symmetry codes: (i) −*x*+1, *y*+1/2, −*z*+1/2; (ii) −*x*+2, *y*+1/2, −*z*+1/2; (iii) −*x*+2, *y*−1/2, −*z*+1/2; (iv) −*x*+1, −*y*+1, −*z*.
